# Profiles of family-focused adverse experiences through childhood and early adolescence: The ROOTS project a community investigation of adolescent mental health

**DOI:** 10.1186/1471-244X-11-109

**Published:** 2011-07-07

**Authors:** Valerie J Dunn, Rosemary A Abbott, Tim J Croudace, Paul Wilkinson, Peter B Jones, Joe Herbert, Ian M Goodyer

**Affiliations:** 1 Developmental and Life-course Research Group, Department of Psychiatry, University of Cambridge, Cambridge UK; 2 Department of Physiology, Development and Neurosciences, Cambridge Centre for Brain Repair, Cambridge UK

## Abstract

**Background:**

Adverse family experiences in early life are associated with subsequent psychopathology. This study adds to the growing body of work exploring the nature and associations between adverse experiences over the childhood years.

**Methods:**

Primary carers of 1143 randomly recruited 14-year olds in Cambridgeshire and Suffolk, UK were interviewed using the Cambridge Early Experiences Interview (CAMEEI) to assess family-focused adversities. Adversities were recorded retrospectively in three time periods (early and later childhood and early adolescence). Latent Class Analysis (LCA) grouped individuals into adversity classes for each time period and longitudinally. Adolescents were interviewed to generate lifetime DSM-IV diagnoses using the K-SADS-PL. The associations between adversity class and diagnoses were explored.

**Results:**

LCA generated a 4-class model for each time period and longitudinally. In early childhood 69% were allocated to a low adversity class; a moderate adversity class (19%) showed elevated rates of family loss, mild or moderate family discord, financial difficulties, maternal psychiatric illness and higher risk for paternal atypical parenting; a severe class (6%) experienced higher rates on all indicators and almost exclusively accounted for incidents of child abuse; a fourth class, characterised by atypical parenting from both parents, accounted for the remaining 7%. Class membership was fairly stable (~ 55%) over time with escape from any adversity by 14 years being uncommon. Compared to those in the low class, the odds ratio for reported psychopathology in adolescents in the severe class ranged from 8 for disruptive behaviour disorders through to 4.8 for depressions and 2.0 for anxiety disorders. Only in the low adversity class did significantly more females than males report psychopathology.

**Conclusions:**

Family adversities in the early years occur as multiple rather than single experiences. Although some children escape adversity, for many this negative family environment persists over the first 15 years of life. Different profiles of family risk may be associated with specific mental disorders in young people. Sex differences in psychopathologies may be most pronounced in those exposed to low levels of family adversities.

## Background

The environments to which our children and adolescents are exposed in their formative years have potentially lasting effects on cognitive and behavioural development. Adversities in these early years are well known to be associated with subsequent psychopathology and consequently have been a focus for mental health researchers for several decades. Studies have used a range of data collection techniques (self-report questionnaires, checklists and comprehensive, semi-structured interviews), definitions and analytic strategies. Despite these disparities across cohorts the empirical association remains robust [1-6] and continues to be the subject of much investigation and a matter for causal speculation.

Many studies have examined the influence of specific adversities, such as parental loss [7] on psychopathology across the life-course. However, concentrating on the effect of any specific adversity in isolation limits our potential understanding of the broader environment and tells us little about other, unmeasured, adversities or protective influences. In the 1970s Rutter and colleagues and Brown and his team advanced the field in the UK by exploring the effects of multiple adversities. Rutter's [8] multifaceted operational index of adversity, made up of severe marital discord, low social and economic class, large family size, paternal criminality, maternal psychiatric disorder and foster care placement, showed strong non-linear associations with mental disorder in childhood. Interestingly a single adversity showed no increased risk but two increased the likelihood of disorder by four times and four predicted a ten-fold increase [9]. Investigating the social causation of affective disorders in female adults Brown and Harris [10] collated experiences of parental indifference, sexual and physical abuse and predicted both depression and anxiety disorders in those with two or more of these childhood adversities (CAs). In the USA National Comorbidity Survey (NCS), 35% of the 8,098 adults reported experiencing three or more CAs in childhood [11] and concluded that CAs commonly occur in clusters rather than individually.

However, by not exploring the relationships of CAs to each other, early studies did little to enhance our understanding of the complex nature of multi-adverse environments. Many simply recorded CAs as present or absent irrespective of their number or independence from each other. Others assumed the predictive value to be in the sum of CAs and grouped individuals based on total scores. Increasingly though investigators are questioning the assumption that quantity confers the greatest risk and are adopting approaches which enable fuller examination of the configurations of CAs [12-16].

A cluster-analysis or person-centred method such as latent class analysis (LCA) [17] is therefore potentially more informative than variable-based techniques. LCA groups individuals according to their patterns of adversity rather than simply the number of CAs reported. From the resultant classes, it becomes apparent which CAs co-occur and how they cluster. The classes can then be related to psychopathology outcomes, or other more proximal risk factors on the pathway to later psychiatric disorders. This understanding of multiple CAs is essential to avoid an inappropriate focus and consequent over estimation of the associations between specific risks and subsequent psychopathology and to better understand the nature and influence of a more complex risk environment.

A number of studies have adopted this approach [14,18-21] and provide evidence that data so analysed reveal relatively consistent clusters (latent class profiles) with different levels of risk for psychopathology. For example Copeland [21], using CA data collected from parents and children in a representative population sample, identified five latent classes: 2 low risk (48.6%), 2 moderate risk (42.8%) and 1 high risk (8.6%). Interestingly the moderate risk classes differed in their prediction of emotional or behavioural disorders whereas the high risk class predicted the highest levels of both. The profiles of childhood adversities in the National Comorbidity Survey Replication Sample suggested that CAs have strong associations with many types of common mental illnesses in adulthood [14,22,23].

The present paper builds on this work by applying latent class analysis to retrospectively recalled indicators of adversity recorded in three phases over the first 15 years of life. The Cambridge Early Experience Interview (CAMEEI), a new developmentally sensitive interview, was used to collect information on adverse experiences from primary carers of adolescents in a large epidemiological cohort study [24]. The aims were to describe clusters of CAs, to generate classes of risk in 3 time periods, to examine continuity of class membership, change in risk over time and to test the clinical validity of the classes by examining associations with DSM IV-defined mental illnesses occurring over the first 15 years of life. We hypothesised that distinct latent classes of risk would index groups of adolescents at differential risk for psychopathology. We also tested the hypothesis that adolescents born to teenage mothers or of lower socio-economic class would be at greater risk for early family adversities.

## Methods

### Sample

ROOTS is a community-based cohort study characterising risk and resilience pathways for emotional and behavioural disorders over the adolescent years. Full details of the theory and methods are described elsewhere [24]. Briefly, we randomly recruited 14 year olds (UK school years 9 and 10) via 18 schools in the Cambridgeshire and Suffolk counties in the East of England. Invitation letters and study information were posted to 3762 families via schools and 1238 (33%) gave written informed consent and entered the study. Of those, 1185 students and 1143 parents proceeded to the interview stage. Adolescents were interviewed in school and parents, usually mothers, were interviewed, predominantly in the family home.

The study was carried out in accordance with the Declaration of Helsinki and Good Clinical Practice guidelines. The study was approved by Cambridgeshire 2 REC, reference number 03/302. At entry into the study all participants and their parents gave written, informed consent.

### Measures

#### 1. The Cambridge Early Experience Interview (CAMEEI)

The CAMEEI, developed specifically for the ROOTS Study, is a researcher-led semi-structured interview which assesses retrospectively exposure to family-based adversities in childhood and adolescence. It is based on the principles of life events and difficulties measurement elucidated by George brown and Tirril Harris (10). In this study, respondents were the primary carers of 14-year olds who provided contextualised information to generate ratings in five domains of the semi-structured interview. The protocol and procedure of the CAMEEI was based on the Newcastle Life Events and Difficulties Schedule developed by one of the authors (IG) [1] and by reference to social inquiry methods focused on adverse experiences in childhood [4]. The interviewers were trained in the semi-structured contextual evaluation procedures by one of the authors (VJD). Panel raters were called when interviewers were uncertain of the face validity of the respondents' descriptions. Pre-interview, parents were posted a set of three timelines corresponding to specific periods in the child's life: (i) early childhood, defined as birth to the start of full-time education, around the age of 5; (ii) later childhood, corresponding to UK primary school years, roughly age 5-11 years; (iii) early adolescence, corresponding to the first 3 or 4 years of UK secondary school, age 11-14. Respondents recorded important events, positive or negative, in the appropriate section of the timeline. At interview, before embarking upon the main, semi-structured set of questions, carers talked researchers through the timelines adding and clarifying details in a conversational, relaxed way until a detailed picture emerged. Timelines were referenced, added to and amended throughout the interview to assist with context and relative timings of events, one to another. This timeline approach has been shown to improve accuracy of recall, the sequential relationship of events and therefore produce a more comprehensive autobiographical narrative of life experiences [25].

The semi-structured section is organized in five domains. Core questions, asked verbatim, are followed by researcher-led discussions based on sets of prompting questions. Firstly presence/absence (p/a) is established and secondly contextual information assesses the negative impact on the family to inform a severity rating of mild, moderate or severe (m/m/s). Approximate durations and ages of onset of the index child are recorded. Items are recorded within each discrete time period to enable tracking over time. Where chronic adversities are reported, for example family discord, it is possible for more than one episode to be reported within a time period. In these cases, all episodes are recorded individually, along with their durations which are then summed for a total duration within that specific time period. A sample page from the CAMEEI showing the family discord question and coding can be found in Additional file 1. The five CAMEEI domains are:

##### Family Relationships

i) family loss and separations (includes step parents and siblings and partners resident for more than 6 months) through divorce, death or adoption (p/a); ii) family discord (m/m/s); iii) lack of maternal affection/engagement with the proband (p/a); iv) maternal parenting style and v) paternal parenting style. The core parenting style question and subsequent discussion is framed to be non-judgemental and to acknowledge parents' differing ideas about parenting styles and punishment regimes: 'Parents have very different ideas about bringing up their children. Thinking about (*each time period*) how strict would you say you (*or partner*) were with (*proband*)?'

Researchers then guide the discussion, gathering examples to build up a contextual picture of the parenting style of each parent. Respondents are then asked to select from a 4-point scale the parenting style which most accurately reflected theirs' and that of their partner for each time period. The scale describes 4 categories - lax, moderate, very strict and cruel-to-be-kind. In circumstances where a respondent's selection conflicts with the picture built up in the discussion, researchers use their discretion to override the respondent to produce a coding which more accurately reflects the parenting style described. Participants are specifically asked 'Did/do you find smacking an effective way of teaching ... a lesson?' Smacking is recorded as never, occasional or regular. The parenting inconsistency item relates to within-parent rather than between-parent unpredictability. Again, researcher-led discussion builds up a long-term, detailed picture to avoid the over-reporting of specific or minor incidents. Inconsistency is coded as absent or present (at least one prolonged period of inconsistent parenting by either parent figure). Due to the low prevalence of some of these items, lax, very strict, cruel-to-be-kind, smacking and inconsistency were combined to form a composite variable for each parent defined as 'atypical parenting' and compared to the moderate parenting category.

##### Family Health

i) lifetime family medical illnesses sufficiently severe to impact on family life (moderate, chronic and life-threatening); ii) lifetime psychopathology in family members is assessed using the MINI Mental State Interview [26] excluding the antisocial disorder section, embedded within the CAMEEI. We amended the MINI to cover all family/step-family members, any partner resident for more than 6 months.

##### Family Economics

i) periods of unemployment (p/a); ii) financial difficulties (m/m/s).

##### Child maltreatment

i) physical abuse; ii) sexual abuse; iii) emotional abuse (p/a). Included here are 'at risk' children defined as those ever having been on the Child Protection Register or for whom there was strong, but inconclusive, evidence of abuse. Prevalence of all types of abuse was low in this sample, so positive responses were combined into a single abuse variable in the analysis.

##### Other events/difficulties

i) criminality among family members (p/a); ii) acute life events (p/a, fire in the home) and iii) chronic social difficulties (p/a, ongoing litigation or the demands of caring for extended family).

The CAMEEI was piloted on eight volunteer mothers who offered advice on wording, content, design and procedure. The consensus of opinion was that the timelines were invaluable both pre-interview to orientate mothers to the time period at their own pace, and as an aid to recall during interview. Mothers also felt the timelines were useful to break the ice and establish rapport at the outset. Our pilot participants recommended that respondents be encouraged to record positive as well as negative events and that we adopt a non-judgemental approach.

#### 2. Adolescent mental state assessment

At entry (aged 14 years) all adolescents were assessed for present and lifetime episodes of psychopathology using sections of the K-SADS-PL (depression, anxiety, eating and behaviour disorders) to generate DSM-IV axis one diagnoses [27]. We designated High Clinical Index (HCI) or 'probable' case status to those who reported significant, impairing symptoms but who fell just short of the full symptom count for disorder. In the K-SADS screen we also recorded non-suicidal self injury (NSSI) defined as any deliberate self-harming or mutilating behaviour (excluding tattoos and piercing) with no suicidal intent. Interviews were conducted by fully trained researchers and diagnoses reached at consensus meetings with senior staff.

#### 3. Other information

A demographic questionnaire recorded maternal age at the birth of the child and a proxy measure of social and economic class using five ACORN categories ranging from wealthy achievers to hard pressed, derived by CACI from post code data (http://www.caci.co.uk).

### Data Analytic Strategy

The aim of our analytic strategy was to develop a summary measure of adversities which captured the relationships between them in the most parsimonious way. We wish to develop an adversity model that indexes the overall differential nature of exposure to multiple adversities over the time course of the CAMEEI interview. This analysis is not focused on prognostic prediction which will be better addressed when examining the putative influence of childhood adversities on the subsequent emergence of mental illnesses in later adolescence.

#### i) Exploratory analysis

Initial data exploration using cross tabulation and correlation analysis, using tetrachoric and polychoric correlations appropriate for these binary or ordinally coded adversity exposures, revealed strong associations. Such empirical associations could be due to latent dimensions of adversity amenable to factor analysis or population sub-groups with different experiences of adversity (adversity profiles). Exploratory and confirmatory categorical factor analysis (suitable for binary and ordinal items) in Mplus 5.1 did not suggest a single unidimensional structure but a more complex pattern with the parenting items loading on a second dimension. Due to only acceptable model fit and only a small number of items loading on the second factor, we opted for a mixture model perspective grouping individuals by their experience of multiple adversities using latent class analysis (LCA), a realistically complex but easy to interpret model.

#### ii) Use of latent class analysis

LCA [17,28] is a model-based clustering technique which enables individuals to be grouped according to their pattern of adversities, rather than the total number experienced. This produces distinct adversity profiles for individuals who would be indistinguishable if grouped by sum score, often used as a proxy measure of severity. Results define the most parsimonious number of classes and their prevalence, whilst also describing the probability of reporting each adversity indicator in each class. Our use of LCA was more exploratory than hypothesis driven in that the optimum number of latent classes was not known a priori. The aim was to find an underlying classification that provided a more reliable summary of the associations in the observed data than that based on summed scores [29].

LCA models make strong assumptions concerning the conditional independence of variables within each latent class. The model requires that the observed variables are uncorrelated once class membership is known. Model parameters were estimated using maximum likelihood (ML). We report the probability of an individual being in a class and the probability of endorsing an adversity indicator within each class. Once the model was identified, posterior probabilities of class membership were based on Bayes theory to allocate individuals to their most likely class.

All models were estimated using Latent Gold version 4.5 (Statistical Innovations, Belmont). We report the log likelihood (LL) for each model and two information criteria, the Bayesian (BIC) and the Akaike information criteria (AIC). Standard chi-square tests of model fit are not appropriate where endorsement of some indicators is low. Therefore we selected our preferred model based on the lowest value of the BIC since this should indicate the most parsimonious solution, taking into account the improvement in model fit that results for a given increase in number of parameters. For model selection it is generally recommended that such statistics are viewed cautiously, in conjunction with interpretation of class profiles, to ensure a meaningful solution is interpreted [17].

#### iii) Analytic procedure

LCA modelling was conducted in three stages for each time period (early childhood, later childhood and early adolescence). Stage 1 involved exploring a series of models using all adversity indicators reported by 1137 respondents (those with missing item level data on more than four variables were excluded, n = 6) and estimating the approximate number of classes required to capture the magnitude of association. This led to the specification of one through five classes for stage 2 using a reduced set of 9 indicator variables. In stage 2, models were refined by combining some of the rarer highly-correlated exposures to increase prevalence to at least 5%, for example abuse with criminality. Acute disturbances, chronic social difficulties, parental and sibling medical illnesses were designated as inactive covariates as they showed negligible model contribution at stage 1. Sibling psychiatric disorder was similarly defined as it proved problematic to distinguish as risk or outcome. Modal class allocations were generated by Latent Gold based on the highest posterior probability rule. These allocations were saved for each time point and then used again in a separate longitudinal latent class analysis, stage 3, to produce a single longitudinal latent class.

We treated exposures that occurred over more than one time-period independently, regardless of previous exposure; we did not adopt a cumulative approach. This enabled us to record change over time and allowed for potential movement from a severe exposure to a milder exposure group. Graphical displays of individual change (or stability) in class allocation over time were produced using the Risk plot command in Stata version 11.1 [30].

Indicator level missing data were included for models in Stages 1 and 2 under a Missing at Random (MAR) assumption as a result of our use of ML estimation. For the final stage, all sample individuals had been allocated a modal class at stage 2. Bootstrap p values (allowing for 1000 random starts) were also used for model selection. Although LCA modelling in Latent Gold allows for partially incomplete data under MAR assumptions, bootstrapping procedures are restricted to complete case models, since bootstrapping cannot imitate the missing mechanism [31].

## Results

A total of 1238 families initially consented to participate in ROOTS and 1143 primary carers (92%) completed the CAMEEI. Non-participants were more likely to be from the moderate/hard pressed ACORN categories (27%) than the sample as a whole (14%; χ^2 ^= 8.8, df = 2, p = 0.01). Of the 1143 interviews, 96% (1092) were biological mothers and 3% (35) biological fathers. The remainder were adoptive mothers (7), both parents (3) and 2 each of extended family members, step-mothers and step-fathers. To assess inter-rater agreement, 48 interviews were observed and independently double-coded. Agreement was high (kappa = 0.7 - 0.9) on those core indicators with sufficient positive endorsements to permit analysis (any family discord, parenting and any financial difficulties).

### Characteristics of the study sample

Gender, ACORN classification and maternal age at birth of the proband were not included in the latent class modelling but used for descriptive purposes only. Of the 1143 adolescents, 622 (54.4%) were females; families were classified as wealthy and urban prosperous (62%), comfortably off (24%) and moderate/hard pressed (14%); 4% of mothers were under 20 at the birth of the proband.

### Prevalence of family adversities

Table 1 shows the prevalence of reported exposures to each indicator of family adversity.

**Table 1:** Characteristics of family adversity indicators from the CAMEEI parent interview (N = 1143)

	Exposure							
	Early childhood		Later childhood		Early adolescence		Any 0-14 years	
	n	%	n	%	n	%	n	%
**Family Relationships**								
1) Family loss (any)*	131	11.5	183	16.0	100	8.8	361	31.6
*Parental Divorce*	119	10.4	166	14.5	94	8.2	333	29.1
*Parental Death*	5	0.4	12	1.1	7	0.6	24	2.1
*Sibling Death*	6	0.5	3	0.3	4	0.4	13	1.1
*Adopted *	7	0.7						
2) Family discord	232	20.5	275	24.4	292	25.8	468	41.2
*Mild (eg. constant tension, lack of warmth)*	108	9.6	138	12.2	167	14.8	288	25.3
*Moderate (eg. major rows, spite, sev.volatility)*	80	7.1	87	7.7	94	8.3	172	15.3
*Severe (eg. violence, fear, abusive)*	44	3.9	50	4.4	30	2.7	73	6.4
3) Father's atypical parenting style	266	23.7	238	21.2	226	20.1	306	27.1
4) Mother's atypical parenting style	104	9.2	83	7.4	108	9.6	140	12.3
5) Lack of maternal affection/engagement	98	8.8	68	6.1	92	8.3	170	15.2
**Family Economic Circumstances**								
6) Periods of unemployment	108	9.8	109	10.0	86	7.8	239	21.2
7) Financial difficulties (any)	172	15.3	159	14.1	129	11.5	302	26.8
*Mild (eg. no outings, holidays, scrimping)*	98	8.7	90	8.0	74	6.6	188	16.7
*Moderate (e.g. debt, mortgage arrears)*	52	4.6	61	5.4	42	3.7	99	8.8
*Severe (eg. Often lack of £ for food)*	22	2.0	8	0.7	13	1.2	36	3.2
**Family Health**								
8) Father psychiatric illness (resid. bio & step)	84	7.7	88	8.0	75	6.7	161	14.3
9) Mother psychiatric illness (resid. bio & step)	178	15.9	201	17.9	188	16.7	352	31.0
10) Sibling psychiatric illness (resid. bio, step, half)	58	5.2	102	9.9	122	10.8	150	13.3
11) Parental medical illness with impact (any)	58	4.7	104	9.2	105	9.3	168	14.8
*Moderate severity & duration (eg. hysterectomy)*	21	1.7	22	1.9	19	1.7	56	4.9
*Chronic (eg. diabetes, disabling back problems)*	22	1.8	47	4.1	50	4.4	62	5.5
*Life threatening (eg. cancer)*	15	1.2	35	3.1	36	3.2	54	4.8
12) Sibling chronic medical illness	18	1.4	27	2.4	27	2.4	37	3.2
**Abuse**								
13) Any abuse, (incl at risk/CPR)	32	2.9	48	4.3	48	4.3	73	6.5
*Sexual (teenage sex not coded)*	1	0.1	6	0.5	11	1.0	13	1.2
*Physical (by adults, not peer bullying)*	18	1.5	12	1.1	10	0.9	22	2.0
*Emotional (eg. Isolation, witness dom. violence)*	27	2.6	42	3.9	32	2.9	57	5.0
*Ever on child protection register (CPR)*	16	1.4					16	1.4
**Family Environment (Other)**								
14) Criminality amongst family members	21	1.8	25	2.2	26	2.3	57	5.1
15) Other acute social disturbances	15	1.3	46	4.1	75	6.6	122	10.7
16) Other chronic social difficulties	94	8.2	126	11.0	192	16.8	276	24.2
**Socio-Demographic Data**								
Sex of Proband (female)					622	54.4		
Acorn Group (moderate/hard pressed)					157	13.7		
Mother - teenage birth	46	4.1						

* Not mutually exclusive. Base N for calculation of %s varies across indicators depending upon missing data (minimum = 1093)

Prevalence is given for each discrete time period to expose changes over the 15 year life-course. Most indicators showed marked consistency in the proportions of individuals exposed at each time period, but parental divorce increased from early (10%) to later childhood (15%) but thereafter dropped (8%) in early adolescence. Exposure to mild family discord, acute life events, chronic difficulties and sibling psychiatric illness peaked in early adolescence.

Tetrachoric correlations of the adversity indicators, together with the descriptive variables for each time period are given in tables 2, 3, 4. The correlations range from -0.26 - 0.78 in early childhood, with similar levels at subsequent time points.

**Table 2:** Tetrachoric correlations of family adversity indicators in early childhood (N = 1143)

		1	2	3	4	5	6	7	8	9	10	11	12	13	14	15	16	17	18
		Family loss	Family discord	Father's atypical parenting	Mother's atypical parenting	Mother lack affection/engagement	Unemployment	Financial difficulties	Father psychiatric illness	Mother psychiatric illness	Sibling psychiatric	Family medical illness	Sibling medical illness	Abuse/at risk	Criminality	.Oth acute disturbances	Oth. chro. difficulties	Acorn group (SES)	Teenage birth
1	**Family loss**																		
2	**Family discord**	0.66																	
3	**Father's atypical parenting**	0.37	0.44																
4	**Mother's atypical parenting**	0.20	0.26	0.63															
5	**Mother lack affection/engagement**	0.25	0.45	0.26	0.44														
6	**Unemployment**	0.30	0.28	0.12	0.17	0.19													
7	**Financial difficulties**	0.48	0.40	0.30	0.14	0.23	0.65												
8	**Father psychiatric illness**	0.36	0.59	0.41	0.24	0.34	0.51	0.46											
9	**Mother psychiatric illness**	0.24	0.40	0.13	0.08	0.56	0.10	0.20	0.20										
10	**Sibling psychiatric illness**	0.47	0.21	0.21	0.09	0.01	0.22	0.22	0.20	0.10									
11	**Family medical illness**	0.13	0.14	0.13	0.18	0.24	0.12	0.05	0.35	0.02	0.17								
12	**Sibling medical illness**	-0.26	0.16	0.07	0.15	0.18	0.33	0.02	0.20	0.26	-0.04	0.28							
13	**Abuse/at risk**	0.53	0.78	0.59	0.43	0.45	0.38	0.48	0.78	0.34	0.16	0.17	0.01						
14	**Criminality**	0.49	0.68	0.41	0.39	0.25	0.41	0.45	0.67	0.34	0.20	0.26	0.39	0.69					
15	**Other acute social disturbances**	0.31	0.23	-0.05	0.09	0.21	0.27	0.09	0.16	0.17	0.10	-0.06	0.14	0.47	0.41				
16	**Other chronic social difficulties**	0.16	0.36	0.16	0.05	0.17	0.12	0.14	0.39	0.18	0.03	0.19	0.07	0.33	0.36	0.51			
17	**Acorn group (SES)**	0.28	0.24	0.23	0.17	0.20	0.24	0.23	0.12	0.16	0.11	-0.24	-0.19	0.15	0.34	-0.01	0.03		
18	**Mother (teenage birth)**	0.38	0.24	0.38	0.00	0.12	0.17	0.28	0.19	0.19	0.24	-0.17	0.06	0.32	0.41	0.11	0.14	0.46	

**Table 3:** Tetrachoric correlations of family adversity indicators in later childhood (N = 1143)

		1	2	3	4	5	6	7	8	9	10	11	12	13	14	15	16	17	18
1	**Family loss**																		
2	**Family discord**	0.66																	
3	**Father's atypical parenting**	0.54	0.51																
4	**Mother's atypical parenting**	0.13	0.31	0.65															
5	**Mother lack affection/engagement**	0.27	0.37	0.27	0.53														
6	**Unemployment**	0.26	0.28	0.14	0.24	-0.02													
7	**Financial difficulties**	0.50	0.62	0.43	0.24	0.20	0.36												
8	**Father psychiatric illness**	0.42	0.68	0.40	0.17	0.29	0.32	0.49											
9	**Mother psychiatric illness**	0.44	0.42	0.14	-0.02	0.30	0.02	0.31	0.25										
10	**Sibling psychiatric illness**	0.16	0.45	0.29	0.01	0.02	0.19	0.34	0.21	0.24									
11	**Family medical illness**	0.12	0.09	0.11	0.09	0.20	0.09	0.21	0.08	0.15	-0.10								
12	**Sibling medical illness**	0.10	0.18	0.19	0.19	0.16	-0.06	0.34	0.16	0.07	0.24	0.10							
13	**Abuse/at risk**	0.54	0.77	0.59	0.47	0.46	0.33	0.46	0.61	0.29	0.37	-0.04	-0.14						
14	**Criminality**	0.29	0.66	0.36	0.28	0.19	0.37	0.35	0.50	0.04	0.38	-0.12	-0.03	0.61					
15	**Other acute social disturbances**	0.47	0.61	0.13	0.31	0.28	0.10	0.23	0.34	0.26	0.14	0.11	0.13	0.53	0.34				
16	**Other chronic social difficulties**	0.12	0.11	0.11	0.17	0.19	0.13	0.08	0.21	0.12	0.12	0.00	0.20	0.08	0.17	0.32			
17	**Acorn group (SES)**	0.22	0.32	0.20	0.20	0.09	0.20	0.30	0.24	0.19	0.19	-0.07	0.09	0.24	0.41	0.22	0.10		
18	**Mother (teenage birth)**	0.30	0.38	0.30	-0.11	-0.20	0.36	0.19	0.23	0.15	0.06	-0.11	-0.01	0.19	0.41	0.26	0.00	0.46	

**Table 4:** Tetrachoric correlations of family adversity indicators in early adolescence (N = 1143)

		1	2	3	4	5	6	7	8	9	10	11	12	13	14	15	16	17	18
1	**Family loss**																		
2	**Family discord**	0.62																	
3	**Father's atypical parenting**	0.32	0.43																
4	**Mother's atypical parenting**	0.04	0.26	0.69															
5	**Mother lack affection/engagement**	0.26	0.61	0.31	0.52														
6	**Unemployment**	0.14	0.11	0.08	0.12	-0.01													
7	**Financial difficulties**	0.34	0.55	0.34	0.18	0.32	0.45												
8	**Father psychiatric illness**	0.51	0.55	0.32	0.17	0.29	0.16	0.53											
9	**Mother psychiatric illness**	0.31	0.43	0.19	0.05	0.27	0.06	0.39	0.28										
10	**Sibling psychiatric illness**	0.24	0.54	0.23	0.03	0.13	0.11	0.49	0.32	0.28									
11	**Family medical illness**	0.06	0.06	0.11	0.08	0.13	0.11	0.19	0.19	0.21	-0.07								
12	**Sibling medical illness**	0.14	0.25	0.23	0.19	0.30	-0.13	0.20	0.14	0.14	0.14	0.09							
13	**Abuse/at risk**	0.36	0.68	0.57	0.38	0.46	0.26	0.45	0.60	0.31	0.38	-0.04	0.12						
14	**Criminality**	0.48	0.60	0.32	0.27	0.30	0.34	0.54	0.49	0.40	0.54	0.02	-0.03	0.58					
15	**Other acute social disturbances**	0.26	0.41	0.14	0.12	0.14	0.07	0.22	0.37	0.24	0.26	0.01	-0.12	0.51	0.41				
16	**Other chronic social difficulties**	0.14	0.27	0.11	0.11	0.29	0.08	0.25	0.21	0.18	0.16	0.01	0.08	0.17	0.25	0.01			
17	**Acorn group (SES)**	0.15	0.29	0.21	0.58	0.16	0.07	0.09	0.30	0.01	0.24	0.15	-0.12	0.02	0.17	0.16	0.11		
18	**Mother (teenage birth)**	0.21	0.15	0.30	-0.01	0.07	-0.03	-0.01	0.21	0.01	0.26	0.07	0.04	-0.02	0.25	0.13	-0.14	0.46	

### Latent class modelling: early childhood to early adolescence

LCA results for models including nine composite adversity indicators are reported from solutions in which up to five classes were estimated and interpreted. The indicators were: i) family loss, ii) family discord, iii) abuse and criminality, iv) financial problems plus unemployment, v) paternal psychiatric illness, vi) maternal psychiatric illness, vii) paternal parenting style, viii) maternal parenting style, ix) maternal lack of affection or engagement. All indicators were modelled as binary (present/absent) with the exception of family discord, which was categorised as i) none, ii) mild, iii) moderate and severe. Log-likelihood values, information criteria and classification accuracy are reported for all models (tables 5 and 6). We judged that four classes provided the most parsimonious solution based on joint consideration of the full range of indices and interpretation of the clusters and class size.

**Table 5:** Early childhood to early adolescence information criteria for latent class models with 1-5 classes

A	LL	BIC(LL)	AIC(LL)	Npar	L^2^	df	p-value	**Class.Err**.
**Early Childhood**								
1-Class	-3955.9	7982.2	7931.8	10	1876.3	1127	<0.001	0.00
2-Class	-3634.9	7417.5	7311.8	21	1234.2	1116	0.008	0.05
3-Class	-3579.8	7384.7	7223.6	32	1124.0	1105	0.340	0.15
**4-Class**	**-3542.1**	**7386.8**	**7170.3**	**43**	**1048.7**	**1094**	**0.830**	**0.11**
5-Class	-3522.7	7425.4	7153.4	54	1009.9	1083	0.940	0.13
**Later Childhood**								
1-Class	-4070.8	8212.0	8161.6	10	2060.1	1127	<0.001	0.00
2-Class	-3687.4	7522.6	7416.8	21	1293.3	1116	<0.001	0.06
3-Class	-3628.0	7481.1	7320.0	32	1174.5	1105	0.072	0.08
**4-Class**	**-3591.0**	**7484.6**	**7268.0**	**43**	**1100.5**	**1094**	**0.440**	**0.15**
5-Class	-3577.0	7534.0	7262.0	54	1072.5	1083	0.580	0.17
**Early Adolescence**								
1-Class	-3909.7	7889.8	7839.4	10	1934.6	1127	<0.001	0.00
2-Class	-3615.1	7377.9	7272.2	21	1345.3	1116	<0.001	0.06
3-Class	-3552.9	7331.0	7169.9	32	1221.0	1105	0.008	0.06
**4-Class**	**-3509.1**	**7320.8**	**7104.3**	**43**	**1133.4**	**1094**	**0.200**	**0.12**
5-Class	-3497.8	7375.5	7103.6	54	1110.7	1083	0.270	0.12

**Table 6:** Assignment probabilities - probabilistic versus modal allocation 4 class model

	Modal Allocation											
	Early Childhood				Later Childhood				Early Adolescence			
Probabilistic Allocation	Low	Moderate	Severe	Atypical parenting	Low	Moderate	Severe	Atypical parenting	Low	Moderate	Severe	Atypical parenting
Low	**0.91**	0.13	0.00	0.00	**0.85**	0.13	0.00	0.02	**0.92**	0.15	0.01	0.02
Moderate	0.08	**0.81**	0.11	0.08	0.14	**0.81**	0.12	0.00	0.07	**0.79**	0.11	0.06
Severe	0.00	0.04	**0.86**	0.01	0.00	0.04	**0.88**	0.02	0.00	0.05	**0.85**	0.03
Atypical parenting	0.01	0.01	0.02	**0.91**	0.01	0.01	0.00	**0.95**	0.01	0.01	0.03	**0.89**

N = 1137. Individuals were assigned to the latent class for which the posterior probability of class membership was highest. Table 6 compares the ratio of the modal predicted (probabilistic) allocation with the modal allocation. High values on the leading diagonal are indicative of good model separation and reflect the quality of the empirical classification. The assignment probabilities for the low adversity group and the atypical parenting group were high; the moderate adversity class was less well discriminated (0.81, 0.81, 0.79). This pattern indicates that those allocated to moderate class also had non-zero probabilities for membership in the low class. Similarly, those allocated to the severe adversity group had non-zero probabilities for membership of the moderate adversity class.

**Figure 1:**
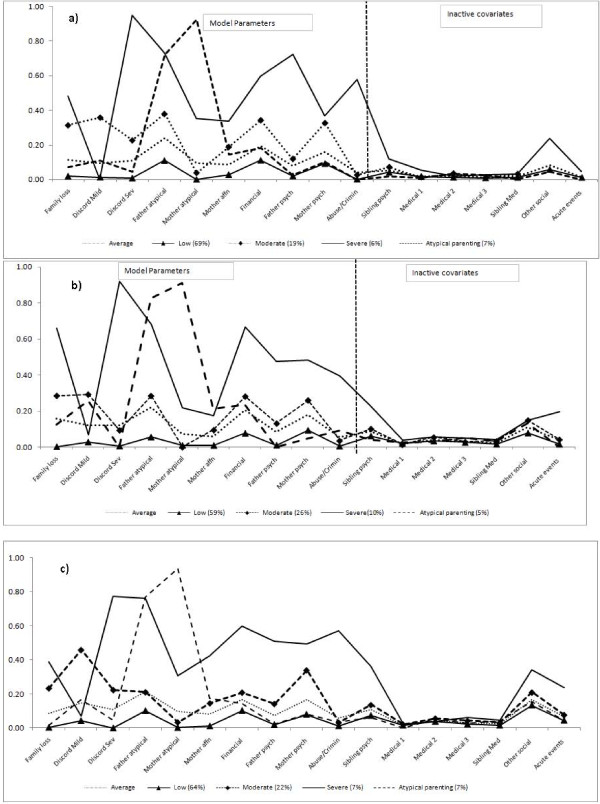
**a: Early childhood, four class model - probability of endorsing exposure by class membership**. b: Later childhood, four class model - probability of endorsing exposure by class membership. c: Early adolescence, four class model - probability of endorsing exposure by class membership. Discord Sev = moderate/severe family discord; father/mother atypical = atypical parenting; mother affn = lack of maternal affection/engagement; financial = financial difficulties unemployment; father/mother psych = primary carers' psychiatric illness; crimin = family criminality; medical = primary carers' medical illness-1 = moderate/severe, 2 = chronic,3 = life threatening.

Figure 1 shows the probability of endorsing each adversity indicator and overall class sizes based on posterior modal allocations. For comparative purposes, the item probabilities of the one class model were plotted to represent the sample average as in table 1.

In early childhood 784 individuals (69%; 436 [56%] females) were allocated to class 1 characterised by a relatively low or zero exposure to any adversities with levels below the sample average. Class 2 comprised 213 individuals (19%; 113 [53%] females) and was characterised by relatively high rates of family loss, mild family discord, paternal atypical parenting, financial difficulties and maternal psychiatric illness. Moderate/severe family discord and maternal lack of affection/engagement were less likely but higher than the sample average. Paternal psychiatric illness was unlikely to be endorsed in this class. Individuals in class 2 were unlikely to have experienced maternal atypical parenting or abuse/criminality. Class 3 consisted of 66 individuals (6%; 31[47%] females) with strikingly high probabilities of moderate/severe family discord (95%), paternal atypical parenting, paternal psychiatric illness. Almost all the abuse/criminality was allocated to this class. The probability of mild family discord in this class was zero. Maternal psychiatric illness was similar to class 2 level, approaching 40%. Compared to other classes, class 3 showed higher probabilities of all other indicators. Finally, 76 individuals were allocated to class 4 (7%; 4 [54%] females) characterised by conspicuously high levels (70%) of atypical parenting from both parents with other indicators close to the sample average except maternal lack of affection/engagement which was slightly elevated but below the levels seen in classes 2 and 3.

Classes 1, 2 and 3 were clearly characterised by severity of exposure. Class probabilities were consistent and distinct across all adversity types. We designated these as low (class 1), moderate (2) and severe (3) adversity classes. In contrast, class 4, characterised almost exclusively by atypical parenting from both parents, was most accurately defined in terms of the nature of the experience rather than severity. This qualitative difference is clear in Figure 1 showing the probabilities of the atypical parenting class membership crossing the 3 severity classes.

The class profiles in later childhood and early adolescence were markedly similar to early childhood. From early through to later childhood there was a rise in the proportion allocated to the moderate class (26% from 19%) and the atypical parenting class (10% from 7%) with a comparatively lower proportion (60% from 69%) allocated to the low class.

Cross-tabulation of class membership with the inactive covariates revealed that chronic social problems, acute disturbances and sibling psychiatric disorder were elevated only in the severe adversity class. Medical illnesses (parental and sibling) were distributed across all 4 classes, with no specific class associations.

Assignment probabilities for the low, severe and atypical parenting classes of the four class model were high (0.85-0.95) (table 6). The moderate class was less well discriminated (0.79-0.81) with these individuals also having non-zero probabilities for membership in the low class.

### Class membership over time

**Figure 2:**
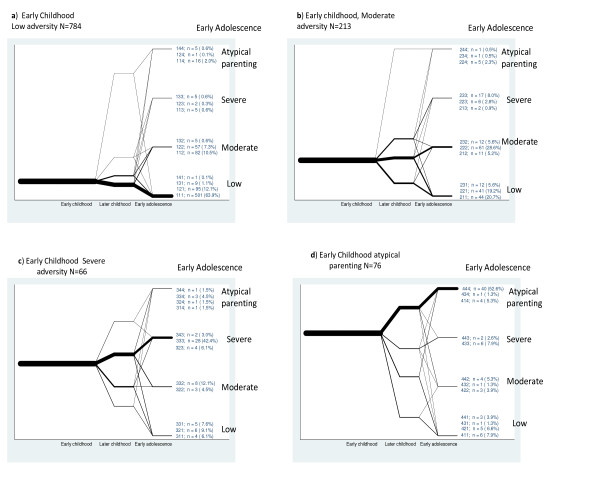
**Pathways of early family environment**.

To understand the extent of mobility between classes over time, pathways were plotted for each class. For ease of presentation, the plots were separated according to class allocation in early childhood. For example, Figure 2a plots the temporal pathways of individuals allocated to the low class in early childhood and similarly, Figure 2b plots the pathways for those starting in the moderate class.

Class membership was fairly stable over time with 55.3% (630) of the total sample remaining in the same class across all time periods (patterns 111, 222, 333, 444).

Stability of class membership over time ranged from 63.9% (501) in the low to 42.4% (28) in the severe and 28.6% (61) in the moderate class. A further small proportion from each class who had switched class in later childhood had reverted to their class of origin by early adolescence. In total 77.2% (606) from the low, 51.5% (45) from the severe and 39.4% (84) from the moderate classes in early childhood were similarly classified in early adolescence.

In a proportion of young people adversity increased over time: 19.9% (156) from the low class in early childhood had been allocated to the moderate or severe class by early adolescence. A further 25 (11.7%) from the moderate class had been elevated to the severe by age 14.

Of those in the moderate class in early childhood, 45.5% (97) had moved out of an adverse environment by early adolescence. In the severe class the figure was 22.8% (15) with a further 16.6% (11) living in a less, though still moderately severe, adverse family environment.

Over half of those in the atypical parenting class (45/76 [59.2%]) in early childhood were similarly classified in early adolescence. Of those 45, 89% (40) remained stable throughout.

### Final model estimation - longitudinal class

Final model estimation computed a longitudinal class based on the modal allocation at each time point. The four class model provided the optimum and most stable solution shown in tables 7 and 8.

**Table 7:** Longitudinal model estimation: information criteria 1 - 6 classes

	LL	BIC(LL)	AIC(LL)	Npar	L^2^	df	p-value	**Class.Err**.	Bootstrap p	se
1-Class	-3354.0	6771.4	6726.1	9	1284.7	54	<0.001	0.00		
2-Class	-2949.9	6033.6	5937.9	19	476.5	44	<0.001	0.04		
3-Class	-2798.6	5801.3	5655.2	29	173.9	34	<0.001	0.05		
**4-Class**	-2728.1	5730.8	5534.3	39	33.0	**24**	**0.100**	**0.08**	**0.186**	**0.02**
5-Class*	-2722.7	5790.3	5543.5	49	22.2	14	0.075	0.08	0.366	0.02
6-Class *	-2718.7	5852.7	5555.5	59	14.2	4	0.007	0.09	0.644	

**Table 8:** Longitudinal model estimation: probabilistic versus modal classification 4 class model

	Modal			
	**Low**	**Moderate**	**Severe**	**Atypical** **parenting**

Low	**0.93**	0.09	0.00	0.01
Moderate	0.06	**0.84**	0.06	0.00
Severe	0.00	0.06	**0.93**	0.01
Atypical parenting	0.00	0.00	0.00	**0.98**

N = 1139 (an additional 2 individuals with missing data in stage 2 were allocated to longitudinal model on basis of available data)

* 5 & 6 class model required 100 starts to avoid local maxima.

The low exposure class (754, 66%) represented individuals with a strong probability of remaining stable throughout, although a small number, allocated longitudinally to this class, had experienced moderate, but not severe, adversity at some point. The longitudinal moderate exposure class (236, 21%) showed more fluctuation over time. Here individuals had a strong probability of being in the moderate adversity class during later childhood, but a lower probability at either early childhood or early adolescence. The severe class (88, 8%) is predominately characterized by persistently high levels of exposure to multiple adversities with moderate/severe family discord and abuse/criminality being prominent. The atypical parenting class (62, 5%) was predominantly characterized by atypical parenting throughout.

Assignment probabilities for the low, severe and atypical parenting classes of the four class model were very high (> = .93). The moderate class was less well discriminated (0.84) with these individuals also having non-zero probabilities for membership in class 1 (table 8).

### Socio-demographic characteristics

There were no significant gender differences in the latent class profiles either at discrete time-points or longitudinally (χ^2^ = 2.7, 3df, p = 0.44). However, there were significant differences according to socio-economic group. The longitudinal severe adversity class comprised 33% of individuals classified as moderate means/hard pressed compared to 10% in the low adversity class and 14% of the overall sample (χ^2^ = 46.3, 6df, p <0.001). The mother's age at the birth of the proband was also differentially represented by the class profiles; longitudinally 13% of the high exposure class were born to teenage mothers compared to 2% in the low, 7% in the moderate adversity classes and 5% in the atypical parenting class (χ^2^ = 26.0, df = 3, p <0.001). These associations are in the expected direction and consistent with prior studies.

### Association of longitudinal class with psychopathology

The lifetime prevalence and differential probabilities of DSM IV diagnoses by longitudinal class, adjusted for gender and ACORN classification, are shown in table 9.

**Table 9:** Association of longitudinal class with psychopathology by age 14

			Longitudinal Latent Classes						
**Diagnoses by age 14**	**N**	**Prevalence**	**Low (66%)**	**Moderate (21%)**		**Severe (8%)**		**Atypical** **parenting (5%)**	

		**%**	**OR**	**OR**	**CI**	**OR**	**CI**	**OR**	**CI**

Any Diagnoses (incl NSSI)	238	21%	1.0	2.3	(1.6, 3.3)	4.0	(2.5, 6.6)	1.4	(0.8, 2.7)
Behaviour disorders (CD, ODD, ADHD)	52	5%	1.0	3.9	(2.0, 7.8)	8.1	(3.8, 17.4)	0.7	(0.1, 5.5)
Affective disorder	91	8%	1.0	2.3	(1.4, 3.9)	4.8	(2.5, 9.2)	1.5	(0.6, 4.1)
Anxiety	71	6%	1.0	1.3	(0.7, 2.4)	1.7	(0.7, 3.7)	0.5	(0.1, 2.3)
Non-suicidal self injury (NSSI) only	94	8%	1.0	1.9	(1.1, 3.1)	2.6	(1.2, 5.3)	1.9	(0.8, 4.8)
Eating disorder	20	2%	n/a						
Substance abuse	7	<1%	n/a						
Alcohol abuse	2	<1%	n/a						

OR Odds ratios adjusted for gender and Acorn classification

Prevalence of eating disorders, substance abuse and alcohol abuse were too low to warrant reliable further analysis

CD = conduct disorder, ODD = oppositional defiance disorder, ADHD = attention deficit hyperactivity disorder.

The odds ratios increased across the three severity classes for psychopathologies. Compared to the low adversity group, severe adversity over time (class 3) was associated with an eightfold increase in odds ratio (OR) for disruptive behaviour disorders (conduct disorder, oppositional defiant disorder and ADHD), a 4.8 times increase for depressive disorders and approaching a two-fold increase for anxiety and non suicidal self injury (NSSI). Exposure to atypical parenting (class 4) suggested an increase in the OR for NSSI and depressive disorders although it should be noted that these associations were not significant by conventional statistical tests. Individuals in the moderate and severe classes were significantly more likely to have had more than 1 diagnosis over their lifetime (11% and 17% respectively) compared to 4% in the low and atypical parenting classes and 7% of the sample as a whole (χ^2^ = 48.8, p <0.001).

### Gender, psychopathology and longitudinal class

We found an interesting association between psychopathology, longitudinal adversity class and gender. In the low adversity class significantly more females than males showed disorder (21% vs 9%, χ^2^ = 22.4, p <0.001). However, this levelled off as severity of adversity increased (moderate class: 35% vs 26%, χ^2^ = 3.7, p = 0.06) until, in the severe adversity class, around 40% of both males and females reported an episode of psychopathology with a significant gender by class interaction (p = 0.02) for the severe class compared to the low adversity class. The atypical parenting class resembled the low class (29% vs 11%, χ^2^ = 3.2, p = 0.07).

## Discussion

In this community cohort of adolescents we interviewed primary carers using the Cambridge Early Experiences Interview to assess family-based adversities in the first 15 years of life. We used latent class analysis to identify common patterns of adversities. We confirmed that individuals clustered into four classes that showed exposure to multiple CAs. This 4-class model using 9 variables derived from 16 adversity indicators was found to be the most parsimonious solution. Three classes of adversity were distinguished by severity and a fourth by qualitatively distinct atypical parenting style not identified in previous studies using these methods.

Although risk factor selection has varied between studies limiting comparisons, our findings resonate with those of Copeland and colleagues [13] where relatively low levels of child physical and sexual abuse were classed within a cluster of severe family discord together with psychiatric illness in parents. Other studies have treated abuse variables as distinct (physical, sexual and emotional) [18] or used a more limited selection of risks [32].

Overall this randomly selected community sample of 14 year old participants showed low levels of adversity. The sample average, represented by the one class model (Figure 1), showed the highest probability of endorsement to be 20% for financial difficulties, paternal atypical parenting and family discord (mild, moderate and severe combined). Other indicators showed around 10% probability except abuse/criminality which was negligible. Interestingly abuse/criminality was confined almost exclusively to the severe adversity class. Our atypical parenting class was distinguished by high levels of atypical parenting in both parents but with a relatively low probability of endorsing other adversity indicators. Compared to the severe class, fathers' parenting deficits were seen at an equally high level whilst mothers' atypical style was more than doubled. This atypical parenting class is novel and has not been identified in previous studies. This class is comprised of parenting indicators, lax, very strict, cruel-to-be-kind, smacking and inconsistency, each of which showed low prevalence within this population. This class may index exposure to a more subtle degree of 'maltreatment risk' than is recorded in the items revealing overt abuse. Crucially it is distinguished from the severe adversity class by its low prevalence of exposure to severe family discord.

Four adversity indicators (acute disturbances, chronic social difficulties, parental and sibling medical illnesses) showed negligible model contribution and were considered as inactive covariates. This implies that any active risk component from these factors was accounted for by other variables. The probability of family medical illness was fairly equally distributed across classes, suggesting that exposure to medical illnesses is qualitatively different from other family environmental risks and should be viewed as distinct.

Our stratification of risk variables into three time periods by using detailed timelines enabled us to describe stability and fluctuation of class membership over the 15 year life-course of our adolescents. Stability of membership in both low and severe adversity classes was striking with over half the total sample remaining in the same class throughout. It is particularly important to note that over half young people allocated to the severe and around 40% to the moderate class in early childhood were similarly placed in adolescence, even though a small proportion had seen a decrease in adversity in the interim. Adversity increased for almost a fifth of those who started out in the low and 12% in the moderate class in early childhood. A more heartening finding was that almost half those who experienced moderate adversity and about a quarter of those from the severe class in early childhood had escaped by early adolescence. Over half the young people exposed to atypical parenting were exposed in all three time periods. Further investigation is required to assess what drives this mobility or stability between classes over time and possible associations with onset and persistence of disorders that emerge later during adolescence and early adulthood.

As we hypothesised, distinct latent classes of family adversity were associated with groups of adolescents at differential risk for psychopathology. The severe class showed the strongest, and the low class the weakest associations. This suggests good discriminant validity of the CAMEEI. In the severe and moderate adversity classes the odds ratio for disruptive behaviour disorders was almost twice that for affective disorders. Non-suicidal self injury (not a formal disorder in DSM IV but a potential DSM V diagnosis) showed less association with adversity than either affective or disruptive behaviour disorders. Interestingly anxiety disorders did not appear to be strongly associated with any adversity class. As these findings are cross-sectional we cannot address the direction of effects between psychopathology and latent class. The lower odds ratios with affective disorders may be explained in part by the young age of our sample (mean 14.5) and the later mean age of onset of depression compared to behaviour disorders.

We found that young people from more financially hard pressed families were over-represented in the longitudinal severe adversity class and were more likely to have been born to teenage mothers. This class showed the greatest risk for psychopathology. This is not a surprising finding and provides further validity for the reporting method and LCA procedure. We did not include these risks in our modeling for methodological reasons. All the adversity indicators entered into our models were recorded across 3 time periods covering 15 years of life which allowed us to account for change over time. The method cannot account for one-off events. Our proxy measure of social class, derived from post code data, could be reliably recorded only at the time of interview. Similarly, maternal age at birth is a stand-alone event.

Interestingly, the well-documented gender difference in the occurrence of mental disorder in young people (females > males) was evident only in the low adversity family environment. As severity increased, so the gender difference declined until similar proportions of females and males experienced disorder in the severe adversity class. This unexpected finding suggests either that males may be more resilient, females more sensitive, or both, in the face of low levels of exposure to family adversities. It is interesting to note that in the atypical parenting class there was a trend for the gender difference to return suggesting that females may be more sensitive to atypical parenting.

These results should be viewed in the light of various limitations. Our retrospective method is limited in comparison to true prospective birth cohort studies [33,34]. Our methods are however more comprehensive than many but a degree of error is inevitable when data is collected retrospectively [35]. Our detailed timelines divided into specific periods, pre-school, primary and secondary school years aided recall, accuracy and allowed mothers to consider the task at their own pace. Many referred to diaries, albums and other family documents for precision and the timelines became detailed narratives, referred to repeatedly for verification during interviews. This method served to minimise the recall bias effects but cannot remove them and some under reporting is likely. We believe the approach has reduced possible telescoping (mis-timing forwards or backwards) and halo effects (where the perception of a specific event as negative or positive infects perception of other events) and there is no evidence of recency effects.

We were further limited by concentrating on family-focused adversities and acknowledge that there may be complex external factors which need to be explored. There are also a number of procedural limitations. When assessing parenting style we combined lack of control, strictness and harsh parenting in a single atypical variable leaving us unable to explore any differential effects of parenting style. Data was collected predominantly from mothers and this may have resulted in the under-or over-reporting of some indicators. The majority of physical and emotional abuse of children occurs at the hands of parents and although sexual abuse by parents is relatively rare, when it does occur, fathers or step-fathers tend to be the main perpetrators [36]. Mothers in such circumstances, therefore, might be unwilling to divulge to a researcher, what would be incriminating information. The current findings show however that such abuse occurs within the context of other multiple adversities and is unlikely to have occurred in the low adversity class where other indicators were rare. Therefore, although abuse may be under-reported this is unlikely to have greatly affected class membership. It is possible that more subtle forms of emotional and physical abuse were not detected. We speculate that these may have been captured in the atypical parenting class which is distinguished by the absence of moderate/severe family discord. Reliance upon maternal report may however have skewed our atypical parenting results in another way. It is quite feasible that mothers minimised their own parenting deficits while over-reporting their partners' shortcomings. However, the defining feature of our atypical parenting class was atypical parenting from both parents indicating the putative lack of such bias. There are also disadvantages to sourcing family psychiatric data exclusively from mothers. The result may be artificially elevated rates of externalising disorders, these being more easily identifiable than internalising disorders. We would argue though that these potential shortcomings are more than compensated for by the detailed overview of family life most mothers are able to provide. As our sample is over-represented in the higher SES groups and predominantly of white ethnic origin the generalisability of our findings is limited and replication is required in other, more diverse settings.

Finally we note that the aim of our data analytic approach was to develop summary measures to account for the interrelationships between family-focused adversities. The 4-class model was the most parsimonious and suggests further disaggregation of particular experiences may be unhelpful. Nevertheless there is a degree of variation in the prevalence of experiences within classes. Our future investigations of the influence of adversities on risk for mental illnesses will aim to determine whether alternative analytic methods (such as regression based models) reveal precise contributions of specific adversities within and perhaps between classes over time.

## Conclusion

The CAMEEI has generated detailed and novel temporal information on family adversities over the first 15 years of life. Applying latent class analysis to this rich data has enabled us to classify individuals exposed to particular clusters of family adversities, map their course over time and identify gender differentiated associations with adolescent psychopathology. The current results highlight the importance of understanding the quality of adverse experience as well as the particular patterning of risks. There is an indication of associations between specific mental disorders and patterns of family risk. Studies of single CAs, such as child sexual abuse, could lead to false causal associations, as they invariably occur amid a more complex constellation of family risk factors which require full understanding.

## Competing interests

The authors declare that they have no competing interests.

## Authors' contributions

VJD drafted the manuscript, designed the CAMEEI, collected data and managed the study. RAA performed all statistical analyses. TJC participated in the design of the study and oversaw the analytical strategy. PW contributed to the analysis. PBJ participated in the design of the study. JH participated in the design of the study. IMG conceived and designed the study, assisted in the design of CAMEEI and contributed at all stages of both the study and manuscript. All authors read, contributed to and approved the final manuscript.

## Pre-publication history

The pre-publication history for this paper can be accessed here:

http://www.biomedcentral.com/1471-244X/11/109/prepub

## Supplementary Material

Additional file 1 **Sample page from the CAMEEI: Family discord question**. The Cambridge Early Experience Interview (CAMEEI) is divided into five domains. This is a sample question used to assess discord in the family. Core questions, asked verbatim, are in bold and these are followed by researcher-led discussions based on sets of prompting questions. Click here for file
