# Supplementary material for: Profiles of family-focused adverse experiences through childhood and early adolescence: The ROOTS project a community investigation of adolescent mental health
Source: BMC Psychiatry. 2011 Jul 7;11:109. doi: 10.1186/1471-244X-11-109 (PMC3199756; doi:10.1186/1471-244X-11-109)
Supplement: Additional file 1 — Sample page from the CAMEEI: Family discord question. The Cambridge Early Experience Interview (CAMEEI) is divided into five domains. This is a sample question used to assess discord in the family. Core questions, asked verbatim, are in bold and these are followed by researcher-led discussions based on sets of prompting questions. [file 1471-244X-11-109-S1.DOC]

**CAMEEI Sample question:**

*(Ask and code for each time period separately)*

**1.** **Have there been times** **when family members have really not got on together?**

*If conflict is reported or suspected, establish family members involved & record in*

*appropriate section on coding sheet. Establish nature and severity of the discord using*

*prompts for reference. Ask specifically about relationships with the participating child.*

-Can you tell me what it was like? What sorts of things would happen? -What was the relationship like?

-How badly did they get on?

-What sort of things provoked an upset?

-Were there raised voices, throwing things, physical violence? Injury? (If so rate severity)

-How often? Daily, weekly, monthly, rarely? How long did this go on for? How old was … when it started?

-Prosecutions? Did anyone end up with a criminal record?

-Did … witness it or were they involved?

-Were any of the children put on the CPR? Were the police or SSD involved?

-In between times, how did they/you get along?

-Did it result in anyone leaving the family? Temporarily/permanently?

**Number** __________

**T1, T2, T3: Age/s of proband:**

**Conflict between:** Parents, siblings, par/sib, prob/par, prob/sib

**Coding guidelines:**

0 - none

1 – mild: some signif icant arguments/tensions but no physical violence. Perhaps little time spent together; some slamming doors; poor communication between family members.

2 - moderate: frequent (at least weekly) serious arguments/tension; very poor communication perhaps long silences; lack of warmth; family members show no interest in each other; perhaps occasional violence or throwing things, but no serious injury.

3 – Severe: daily (or near daily) severe disagreements/tension. Regular violence, frequent breakages or intense arguments. Complete breakdown in communication.

**CPR:** 0 – No 1 – Yes

**Practical impact on daily life**:

0. little/no significant

1. significant impact on family life. Examples may be: separation; parent/s may have struggled to keep household going or suffered depression or anxiety response, daily care of children suffered, children may have missed school or left to own devices, unsupervised or spent some time living elsewhere.

**Duration (months): Current:** 0-no 1-yes

NB: Did this result in health problems (q.9, 10, 1, 12); housing problems (q.16, 1); school problems (q.20-23); unemployment/financial problems (q.13, 14).
